# Analysis of BCLI, N363S and ER22/23EK Polymorphisms of the Glucocorticoid Receptor Gene in Adrenal Incidentalomas

**DOI:** 10.1371/journal.pone.0162437

**Published:** 2016-09-20

**Authors:** Giuseppe Reimondo, Iacopo Chiodini, Soraya Puglisi, Anna Pia, Valentina Morelli, Darko Kastelan, Salvatore Cannavo, Paola Berchialla, Daniela Giachino, Paola Perotti, Alessandra Cuccurullo, Piero Paccotti, Paolo Beck-Peccoz, Mario De Marchi, Massimo Terzolo

**Affiliations:** 1 Internal Medicine 1, Department of Clinical and Biological Sciences, University of Turin, San Luigi Gonzaga Hospital, Orbassano, Italy; 2 Unit of Endocrinology and Metabolic Diseases, Fondazione IRCCS Cà Granda-Ospedale Maggiore Policlinico, Milano Department of Clinical Sciences and Community Health, University of Milan, Milan, Italy; 3 Endocrine Unit, Department of Clinical and Experimental Medicine, University of Messina, Policlinico G. Martino Hospital, Messina, Italy; 4 Department of Endocrinology, University Hospital Zagreb, Zagreb, Croatia; 5 Statistical Unit, Department of Clinical and Biological Sciences, University of Turin, San Luigi Gonzaga, Hospital, Orbassano, Italy; 6 Medical Genetics, Department of Clinical and Biological Sciences, University of Turin, San Luigi Gonzaga Hospital, Orbassano, Italy; University of Birmingham, UNITED KINGDOM

## Abstract

**Context:**

Patients with adrenal incidentalomas (AI) may experience detrimental consequences due to a minimal cortisol excess sustained by adrenal adenoma. SNPs of the glucocorticoid receptor gene (*NR3C1*) modulate individual sensitivity to glucocorticoids and may interfere with the clinical presentation.

**Objective:**

To compare the frequency of N363S, ER22/23EK and BclI SNPs in patients with AI with the general population and to evaluate whether these SNPs are linked to consequences of cortisol excess.

**Setting:**

Multicentric, retrospective analysis of patients referred from 2010 to 2014 to 4 centers (Orbassano, Milano, Messina [Italy] and Zagreb [Croatia]).

**Patients:**

411 patients with AI; 153 males and 258 females and 186 from blood donors.

**Main outcomes measures:**

All patients and controls were genotyped for BclI, N363S and ER22/23EK and SNPs frequency was associated with clinical and hormonal features.

**Results:**

SNP frequency was: SNP frequency was: N363S 5.4% (MAF 0.027), BclI 54.7% (MAF 0.328), ER22/23EK 4.4% (MAF 0.022), without any significant difference between patients and controls. N363S was more frequent in hypertensive patients (p = 0.03) and was associated with hypertension (p = 0.015) in patients with suppressed cortisol after the 1-mg DST.

**Conclusions:**

Our results demonstrate that SNPs of the glucocorticoid receptor gene do not play a pathogenetic role for AI. The impact of any single SNP on the phenotypic expression of minimal cortisol excess is limited and their analysis does not provide additional data that may be exploited for patient management.

## Introduction

Several glucocorticoid receptor (GR) gene polymorphisms have been described that may play a crucial role for glucocorticoid action, influencing glucocorticoid sensitivity and conferring a potential susceptibility to a variety of diseases, such as osteoporosis [1] or metabolic disorders [2, 3]. However, different GR single nucleotide polymorphisms (SNPs) show varying functional significance. The BclI and the N363S SNPs have been associated in the general population with enhanced glucocorticoid sensitivity and, consequently, with increased abdominal fat mass, higher cholesterol levels, hyperinsulinemia, and low bone mineral density [4, 5, 6, 7]. In contrast, the ER22/23EK GR SNP is associated with relative GC resistance that may confer to carriers favorable metabolic parameters and body composition [3, 8].

Investigation of GR gene SNPs in patients with Cushing’s syndrome has shown a higher frequency of BclI compared with healthy controls, while no significant differences were found in the frequency of the other SNPs. Despite the significantly increased levels of morning serum cortisol in the BclI carriers, no clinical or metabolic differences were found between these patients and wild type carriers [9]. In another study, patients with Cushing’s syndrome with the BclI SNP had reduced femoral bone mineral density compared to patients with wild type GR[1].

In patients with Addison’s disease, Giordano at al. found that BclI may contribute to increased central adiposity, impaired glucose metabolism and dyslipidaemia [10]. Another study suggests that the homozygous BclI genotype (GG) may be associated with higher bone resorption in adult with primary adrenal insufficiency. Moreover, GG carriers needed lower hydrocortisone replacement doses supporting the view that this GR SNP is associated with increased cortisol sensitivity[11].

Patients with adrenal incidentalomas are an interesting cohort to study GR SNPs, since 5% to 30% of such patients may show mild cortisol excess without specific Cushingoid signs [12, 13]. However, chronic exposure to an even minimal cortisol excess is not exempt of detrimental consequences. Evidence is gathering that this condition portends an increased risk of cardiovascular events and an impaired bone health with high fracture risk [14,15]. Thus, it is tempting to speculate that the individual sensitivity to glucocorticoids may play a role to influence development of the classic complications of cortisol excess in patients exposed to slightly elevated cortisol levels.

The aims of the present study were to assess whether the prevalence of N363S, ER22/23EK and BclI SNPs is different in patients with adrenal incidentalomas compared to the general population, and to evaluate whether these SNPs may be linked to any hormonal or metabolic abnormalities that confer an increased cardiovascular risk in patients with adrenal incidentalomas.

## Patients and Methods

### Study subjects

We did a retrospective evaluation of 411 patients (median age 62 years, range 33–86 years). They were 153 male (median age 63 years, range 35–86 years) and 258 female (median age 61 years, range 33–84 years) subjects, who were drawn from a series of consecutive patients with adrenal incidentalomas referred to four centres partaking in the European Network for the Study of Adrenal Tumours from 2010 to 2014 (Orbassano, Milano, Messina [Italy] and Zagreb [Croatia]).

In agreement with the definition of adrenal incidentaloma (adrenal mass detected unexpectedly by imaging procedures performed for reasons unrelated to adrenal diseases) [12] patients with severe or resistant hypertension, or paroxysmal hypertension, or hypokaliemia, or clinical signs of hypercortisolism (facial plethora, striae rubrae, easy bruising, proximal muscle weakness) were excluded. Patients with known extra-adrenal malignancies were also excluded from the study. From the overall series of adrenal incidentalomas, only the patients with a presumed cortical adenoma were selected for the study.

Diagnosis of adrenal adenoma was based on the following CT features: size less than 6 cm, regular mass shape with well-defined margins, and homogeneous enhancement with attenuation value less than 10 Hounsfield Units on unenhanced CT [12, 16, 17]. In all patients, CT was repeated after 3 to 6 months to document unchanged CT features. Catecholamine excess and primary aldosteronism were excluded in all patients by measuring urinary fractionated metanephrines, and by paired evaluation of PRA and plasma aldosterone concentration. A group of 186 healthy volunteers (median age 60 years, range 35–82 years) recruited from blood donors served as controls. The study was performed according to the Helsinki Declaration II with written informed consent obtained from all subjects and approved by the Local Ethics Committees of the San Luigi Gonzaga Hospital.

For all subjects, weight, height, BMI, systolic blood pressure (SBP), and diastolic blood pressure (DBP) were evaluated. A BMI above 30 kg/m^2^ was considered as an index of obesity [18]. The waist was measured as the minimum abdominal circumference between the xiphoid process and the umbilicus; waist conference above 88 cm in women and 102 cm in men defined central obesity [19, 20]. According to the ESH and ESC 2013 guidelines [21], blood pressure was measured in the non-dominant arm, with subjects in a relax sitting position, using a mercury sphygmomanometer placed at the heart level; the average of three measurements was calculated. Hypertension was diagnosed when SBP values were >140 mmHg and/or DBP values >90 mmHg, or whether anti-hypertensive treatment was instituted. Diabetes mellitus was diagnosed when fasting blood glucose levels were 7 mmol/L or greater in two consecutive determinations or at least 11.1 mmol/L 2h after an oral glucose load [22, 23] or in patients on insulin or hypoglycemic agents. Hypertriglyceridemia was diagnosed when triglyceride levels were above 1.69 mmol/L, whereas hypercholesterolemia was diagnosed when LDL cholesterol levels were above 4.13 mmol/L, and low HDL cholesterol levels were below 1.03 mmol/L [19, 20].

### Biochemical tests

Fasting glucose, triglycerides, and total, LDL, HDL cholesterol were measured by standard procedures.

All patients underwent measurements of morning ACTH, 24 -h urinary free cortisol (UFC) and serum cortisol after overnight 1 -mg dexamethasone suppression test (DST). Premenopausal women were studied in the early follicular phase of the menstrual cycle. Post-DST cortisol levels were used to stratify patients in three groups: group 1, patients with complete suppression (cortisol ≤1.8 μg/dL, 50 nmol/L); group 2, patients with incomplete suppression (cortisol between 1.9 μg/dL and 3.0 μg/dL [52–83 nmol/L]); group 3, patients with overtly inadequate suppression (cortisol >3.0 μg/dL [83 nmol/L]). Hormonal variables were measured in a single laboratory for each center using commercially available reagents. All samples for an individual subject were determined in a single assay in duplicate. Intra- and inter-assay coefficients of variation for all hormone variables were less than 8% and 12%, respectively.

### SNP analysis

All patients and controls were genotyped for the presence of BclI, N363S and ER22/23EK SNPs.

Total genomic DNA was isolated from 400 μL peripheral blood collected in EDTA tubes using the automatic extractor “Maxwell”(Promega Corporation, Madison, WI, USA). The *NR3C1* gene SNPs were studied by a pyrosequencing assay (Qiagen, Hilden, Germany): BclI (rs41423247), N363S (rs56149945) and ER22/23EK (rs6189 and rs6190) as described in Giordano et al. [10] on a PyroMark Q96MA apparatus (Qiagen, Hilden, Germany). Primers and amplification condition are listed in Table 1.

**Table 1:** PCR and pyrosecquencing primer.

NR3C1 SNPs	Forward Primer	Reverse Primer	Pyrosequencing Primer	TA PCR	Amplicon lenght
ER23/23EK	Bio 5’-GAAGAAAACCCCAGCAGTGTG -3’	5’-GACGCAGAAACCTTCACAGTAGCT -3’	5’-GGTTTTATAGAAGTCCATCA-3’	61°C	98bp
N363S	5’- ACAGCAGGATCAGAAGCCTATTT -3’	Bio 5’-AGTTCAGAGTCCCCAGAGAAGTC -3’	5’- CCGTTGGTTCCGAAA -3’	57°C	119bp
BclI	5’- AGGTCTTGCTCACAGGGTTCTTG -3’	Bio 5’- GAACTTGCAGGAACATTTGAACG -3’	5’- AAGTAGACAAGTTATGTCTG -3’	58°C	128bp

Bio: the 5’ biotin modification of the PCR primer is required for the DNA single strand DNA capture in the Pyrosequencing assay protocol.

TA: annealing temperature for Polimerase Chain Reaction

### Statistical Analysis

Continuous data were expressed as mean, standard deviation and interquartile range, while categorical variables were given as counts and percentages. Comparison between groups was made using the Mann-Whitney U test for continuous variables and the χ2 test for categorical variables.

For each SNP, the association with the evaluated variables was performed by logistic regression models adjusting for potential confounders. Statistical backward selection procedure was applied to retain in the model all relevant variables that significantly improved the goodness of fit on the basis of the Akaike Information Criterion (AIC). Interaction among variables was tested by the Wald’s χ2 test. The Firth’s correction was applied to reduce bias in the estimates, due to the small number of events [24]. Statistical analyses were carried out using R version 3.0.2 [25]. Levels of statistical significance were set at p less than 0.05.

## Results

In the overall series, 65.2% of patients had hypertension, 75.4% were obese, 28.4% had diabetes and 44.8% had an altered lipid profile. The patients with a normal post-dexamethasone suppression were 55.7%, while 21.9% had cortisol after DST higher than 3 μg/dL (83 nmol/L) and 22.4% of patients had post-dexamethasone cortisol levels between 1.9 μg/dL and 3.0 μg/dL (52–83 nmol/L). The clinical and biochemical characteristics of the patients are reported in Table 2.

**Table 2:** Clinical and biochemical characteristics and allelic frequency of the patients with adrenal incidentalomas categorized for cortisol secretion after the 1 mg DST.

	Group 1	Group 2	Group 3	
	N = 229	N = 92	N = 90	
**Age (years)**	59.7 ± 10.1	65.1 ± 8.6	63.2 ± 9.5	1 vs 2, p<0.001; 1 vs 3, p = 0.005; 2 vs 3, p = 0.15
**Sex (% women)**	64.9	60.3	66.7	1 vs 2, p = 0.4; 1 vs 3, p = 0.7; 2 vs 3, p = 0.4
**BMI >25 Kg/m2 (%)**	79.1	73.2	72.7	1 vs 2, p = 0.2; 1 vs 3, p = 0.2; 2 vs 3, p = 0.9
**Elevated waist (%)**	55.5	50.0	51.3	1 vs 2, p = 0.4; 1 vs 3, p = 0.5; 2 vs 3, p = 0.9
**Hypertension (%)**	59.7	69.0	72.8	1 vs 2, p = 0.1; 1 vs 3, p = 0.03; 2 vs 3, p = 0.6
**Diabetes (%)**	24.0	28.4	36.6	1 vs 2, p = 0.4; 1 vs 3, p = 0.02; 2 vs 3, p = 0.2
**Dyslipidemia (%)**	41.6	48.8	46.8	1 vs 2, p = 0.2; 1 vs 3, p = 0.8; 2 vs 3, p = 0.4
**UFC (% ULN)**	8.7	16.5	15.3	1 vs 2, p = 0.04; 1 vs 3, p = 0.08; 2 vs 3, p = 0.8
**ACTH (pg/ml)**	17.9 ± 16.4	12.9 ± 8.8	9.2 ± 6.8	1 vs 2, p = 0.006; 1 vs 3, p<0.001; 2 vs 3, p = 0.001
**N363S % (n) (MAF)**	4.8% (11) 0.023	5.4%(5) 0.034	6.6% (6) 0.028	1 vs 2 p = 0.6, 1 vs 3 p = 0.8, 2 vs 3 p = 0.8
**BclI % (n) (MAF)**	53.2% (122) 0.321	56.5% (52) 0.346	53.3% (48) 0.322	1 vs 2 p = 0.5, 1 vs 3 p = 0.9, 2 vs 3 p = 0.7
**ER22/23EK % (n) (MAF)**	5.2% (12) 0.026	3.2% (3) 0.021	3.3% (3) 0.017	1 vs 2 p = 0.8, 1 vs 3 p = 0.6, 2 vs 3 p = 0.8

GROUP 1 = Cortisol ≤ 1.8 mcg/dL

GROUP 2 = Cortisol >1.9 mcg/dL and ≤3 mcg/dL

GROUP 3 = Cortisol >3 mcg/dL

ULN = Upper limit of normality

Continuous variable are expressed as mean ± SD

To convert conventional units to SI units: for cortisol, multiply by 27.59 to obtain nanomoles per liter; for ACTH, multiply by 0.22 to obtain picomoles per liter.

The allelic frequencies of the BclI, N363S and ER22/23EK SNPs were in Hardy-Weinberg equilibrium. The carrier frequency for the three variants in patients with AI were as follows, N363S 5.4% (MAF 0.027), BclI 54.7% (MAF 0.328), ER22/23EK 4.4% (MAF 0.022). We did not observe any significant difference between patients and controls as to the SNP carrier frequency (5.4% vs 9.1% (MAF 0.046) for N363S, p = 0.1, 54.7% vs 55.4% (MAF 0.309) for BclI, p = 0.9 and 4.4% vs 3.8% (MAF 0.019) for ER22/23EK, p = 0.7). Italian (n = 310) and Croatian (n = 101) AI patients were comparable either as carrier frequencies (4.5% vs 7.9% for N363S, p = 0.2, 53.2% vs 59.4% for BclI, p = 0.3 and 3.9% vs 5.9% for ER22/23EK, p = 0.4) or as MAF (0.023 vs 0.04 for N363S, p = 0.4, 0.318 vs 0.361 for BclI, p = 0.4 and 0.019 vs 0.03 for ER22/23EK, p = 0.5). The same was true when patients were stratified for cortisol levels after DST (Table 2). We have not observed any significant difference between our data and the available frequency in the European population (p = 0.3 vs EUR, p = 0.7 vs EA and p = 0.7 vs NFE for N363S, p = 0.07 vs EUR for BclI, p = 0.4 vs EUR, p = 0.4 vs EA and p = 0.7 vs NFE for ER22/23EK) (Table 3).

**Table 3:** Tabular overview of all NR34C1 variants reported in this paper.

			This study	MAF in control population		
Current name	Change at DNA and protein level	refSNP	MAF (411 patients)	1000 Genomes EUR (NCBI Assay ID)	ESP EA	ExACNFE
ER23/23EK	c.66G>A (p.Glu22Glu)	rs6189	2.19%	2.98% (ss1317539291)	2.93%	2.44%
	c.68G>A (p.Arg23Lys)	rs6190		2.98% (ss1317539290)	2.85%	2.44%
N363S	c.1088A>G (p.Asn363Ser)	rs56149945 (former rs6195 or rs356601909)	2.67%	1.79% (ss1317539264)	3.06%	3.19%
BclI	c.1184+646C>G (p.?)	rs41423247	32.8%	37.97% (ss1317539254)	N/A	N/A

**MAF**: Minor Allelic Frequency

**1000 Genomes**: 1000 genome project Phase3_V1 A global reference for human genetic variation, The 1000 Genomes Project Consortium, Nature 526, 68–74 (01 October 2015) doi:10.1038/nature15393. (URL: http://www.1000genomes.org/) [07,2016 accessed].

**EUR**: 1,006 samples of European ancestry

**ESP**: Exome Variant Server, NHLBI GO Exome Sequencing Project (ESP), Seattle, WA (URL: http://evs.gs.washington.edu/EVS/) [07,2016 accessed]

**EA**: 4,300 European American individuals

**ExAC**: Exome Aggregation Consortium (ExAC), Cambridge, MA (URL: http://exac.broadinstitute.org) [07,2016 accessed]

**NFE**: al least 33,329 European (Non Finnish) subjects

**N/A**: not available, deep intronic position

A higher prevalence of N363S was observed in hypertensive patients (p = 0.03) and dyslipidemic patients (p = 0.06), while we did not find the any association for BclI and ER22/23EK (Table 4). We did not find any difference in hormonal data between wild type subjects and SNP carriers (Table 5). In univariate analysis, hypertension was associated to older age (p<0.001), higher BMI (p<0.001), higher cortisol levels after DST (p = 0.005) and presence of the N363S SNP (p = 0.03) (Table 6). In a multiple regression analysis in the whole series including age, sex, BMI and the N363S SNP, hypertension was associated to sex and BMI (p = 0.02 and p<0.01, respectively) while age and N363S SNP were at the limit of the statistical significance (p = 0.06). The N363S SNP was found to be independently associated with hypertension (p = 0.015) in patients with suppressed cortisol after the 1-mg DST (Table 7). In univariate analysis diabetes was associated with sex (p<0.001), age (p = 0.01) and cortisol levels after the 1-mg DST (p = 0.02).

**Table 4:** Correlation between clinical variables and the N363S, Bcl1, ER22/23EK haplotypes, in patients with adrenal incidentalomas.

	N363S			Bcl1			ER22/23EK		
	Wild type	Hetero-homozygote	p value	Wild type	Hetero-homozygote	p value	Wild type	Hetero-homozygote	p value
Obesity	72.5% (282/389)	81.8% (18/22)	0.4	75.7% (143/189)	70.7% (157/222)	0.3	73.3% (288/393)	66.7% (12/18)	0.7
Hypertension	63.8% (248/389)	86.4% (19/22)	0.03	68.3% (129/189)	62.1% (138/222)	0.2	64.1% (252/393)	83.3% (15/18)	0.1
Diabetes	25.2% (98/389)	31.8% (7/22)	0.6	23.8% (45/189)	27.0% (60/222)	0.5	24.9% (98/393)	38.9% (7/18)	0.3
Dyslipidaemia	43.2% (168/389)	63.6% (14/22)	0.06	45.5% (86/189)	43,2% (96/222)	0.9	44.0% (173/393)	50% (9/18)	0.8

**Table 5:** Hormonal features in the different GR polymorphic variant.

	N363S			Bcl1			ER22/23EK		
	Wild type (n = 389)	Hetero-homozygote (n = 22)	p value	Wild type (n = 189)	Hetero-homozygote (n = 222)	p value	Wild type (n = 393)	Hetero-homozygote (n = 18)	p value
Cortisol post DST (mcg/dL)	2.4 ± 2.0	2.5 ± 1.7	0.8	2.5 ± 2.2	2.3 ± 1.9	0.3	2.4 ± 1.9	2.7 ± 2.6	0.5
ACTH (pg/mL)	14.5 ± 13.0	17.7 ± 12.1	0.3	16.3 ± 14.3	14.4 ± 8.1	0.09	14.2 ± 12.1	17.5 ± 14.7	0.3
UFC (% ULN, n.)	12.8 (50)	9.0 (2)	0.7	9.5 (18)	15.3 (34)	0.1	12.2 (48)	22.2 (4)	0.3

Data are expressed as mean ± SD with the exception of UFC expressed as percentage of patients with levels higher than the upper limit of normality (ULN) for the reference laboratory.

To convert conventional units to SI units: for cortisol, multiply by 27.59 to obtain nanomoles per liter; for ACTH, multiply by 0.22 to obtain picomoles per liter.

**Table 6:** Correlation between the dependent variable hypertension with clinical, hormonal data and GR polymorphism in univariate analysis.

	patients without hypertension N = 143	patients with hypertension N = 268	p value
**sex (female)**	59%	65%	0.3
**age (years)**	53/60/66	57/64/71	<0.001
**BMI >25 Kg/m2**	64%	81%	<0.001
**Cortisol after DST (mcg/dL)**	1.1/1.7/2.4	1.4/2.0/3.0	0.005
**N363S (MAF)**	2% (0.01)	7% (0.044)	0.03
**BclI (MAF)**	58% (0.336)	53% (0.324)	0.3
**ER22/23EK**	2% (0.01)	6% (0.03)	0.09

Continuous variables are reported as I quartile/median/ III quartile

To convert conventional units to SI units: for cortisol, multiply by 27.59 to obtain nanomoles per liter

**Table 7:** Multiple regression analysis with Firth correction.

	AGE	SEX	BMI	N363S
**HYPERTENSION (whole series, n = 267)**	p = 0.06	p = 0.02	p<0.01	**p = 0.06**
**HYPERTENSION (DST <1.8 mcg/dl, n = 137)**	p = 0.05	p = 0.02	p<0.01	**p = 0.015**

R^2^: 0.06; p< 0.01

## Discussion

The first aim of the present study was to assess the frequency of the most studied GR SNPs in the largest ever published series patients with adrenal incidentalomas.

Previous studies describe a wide range of prevalence for each SNPs depending on ethnicity. In the European population, the frequency of hetero/homozygote variant for N363S is estimated to be 3–9%, BclI 35–46% and ER22/23EK 1.6–9% [3, 4, 8, 26]. With the exception for a non-significant increase of BclI in the patients group (54.7%), we have confirmed previously published estimates in the general population.

There are studies claiming that some GR variants may have a pathogenetic role on tumour growth or cortisol production. In a previous study, the C allele of BclI and a minor allele of 9β were associated with the presence of adrenal incidentaloma[27]. Tzanela and coll. [28] genotyped 95 subjects with adrenal incidentaloma for the BclI SNP reporting smaller size of adrenal nodules in patients carrying the variant. Majnik et al. [29] found that the frequency of the N363S variant was markedly higher in patients with bilateral incidentalomas, suggesting a possiblepathogenetic role. Trementino and coll.[9] reported a higher prevalence of BclI variants in patients with Cushing’s syndrome. Szappanos did not confirm these data, demonstrating that BclI, N363S, ER22/23EK and A3669G (rs6198, c.*3833A>G) did not influence the development of Cushing’s disease, or adrenal dependent Cushing’s syndrome [1]. Notwithstanding that the assumption of a pathogenetic role for the GR SNPs is based only on a presumed difference in their prevalence and is not supported by functional studies, we did not observe any difference in the SNP prevalence between patients and controls. Discrepancy with some previous studies may be accounted for the limited sample size of such studies.

The second aim of the study was to assess the genotype to phenotype correlation of BclI, N363S, and ER22/23EK SNPs of the GR gene in patients with a adrenal incidentalomas. Adrenal incidentalomas represent an interesting model being characterized by an individual variability of the cortisol secretory pattern along a continuum from normal cortisol secretion to cortisol autonomy, in the absence of a specific Cushingoid phenotype. In our series, approximately 45% of patients showed some degree of cortisol autonomy, heralded by incomplete to grossly reduced cortisol suppression following the 1-mg DST. The 1-mg DST has been traditionally used to explore the integrity of the pituitary feedback and is very sensitive for detecting autonomous cortisol secretion.

The evaluation of the influence of GR polymeric variants on metabolic profile and cardiovascular risk has been extensively studied in different clinical settings but results are not homogeneous. Attention has been focused on N363S and BclI SNPs, which theoretically confer enhanced sensitivity to glucocorticoids giving a worsen metabolic profile. However, many studies did not observe any correlation between the different SNPs and the metabolic profile or cardiovascular risk [26, 30, 31, 32]. Conflicting results have been also reported for the ER22/23EK polymorphism in general population. Some studies demonstrated higher cortisol levels after dexamethasone suppression associated to increased insulin sensitivity, better metabolic profile and reduced weight, although with a striking gender difference in the SNP effect [3, 8, 33, 34]. Other studies did not confirm these data [26, 35].

In patients with Addison’s disease, a small study on 4 patients carrying the homozygous BclI polymorphism GG showed a significant BMI increase and a worsen metabolic profile [10]. Ross and coll.[36] showed similar findings, however the analysis was not corrected for the hydrocortisone dose. A more recent paper by the same authors [37] performed with a rigorous statistical evaluation has not demonstrated any influence of the 9β polymorphism on hydrocortisone dose and clinical consequences.

Also in patients with Cushing’s syndrome, available data do not support the view that SNPs variants of GR gene may modulate the effects of cortisol on peripheral tissues. Trementino et al. [9] reported a higher frequency of the BclI SNP in patients with Cushing’s syndrome compared to healthy subjects, without any clinical or metabolic consequences in patients carrying the polymorphism. Moreover, SNPs were not associated with fracture risk. Conversely, Szappanos et al. [1] suggested that BclImay modify the skeletal sensitivity to the glucocorticoid excess in the 6 patients of the entire series carrying the polymorphic variant.

Scanty data are available in patients with adrenal incidentalomas. Studies have a limited statistical power, since they usually report on less than 10 patients with the N363S variant, due to the reduced frequency of the SNP in the general population. The present study represent the largest series of patients with incidentally discovered adrenal adenoma genotyped for the presence of BclI, N363S and ER22/23EK SNPs. The clinical characteristics of our patient do not differ with those previously published, confirming the high frequency of hypertension, diabetes and cortisol autonomy in such patients. Our results demonstrate that the patients carrying BclI, N363S and ER22/23EK SNPs do not present any significant hormonal characteristics compared to the wild-type patients for the GR gene. These results confirm a previous paper by Morelli showing that the single polymorphic variant did not influence cortisol secretion [14].

As to the issue of whether the GR SNPs may modulate patient presentation, we found that the N363S polymorphism may be correlated with the presence of hypertension, at least in the group of patients who normally suppress cortisol after the 1-mg DST. No phenotype correlation was found with the other studied SNPs. Thus, we confirm and extend results of previous studies on a small number of patients stratified into two groups by the presence of the sensitizing SNPs, which were associated with increased prevalence of arterial hypertension and vertebral fractures. It is possible to speculate that the relationship between the N363S SNP and the risk of hypertension may be masked when a minimal degree of cortisol excess becomes apparent.

We should acknowledge the limits of a retrospective, multicentre evaluation and the lack of data on bone metabolism and vertebral fracture. However, the patients were studied in referral centres for adrenal disease where pre-specified specific protocols have been set. We are also aware that the sensitivity of the glucocorticoid receptor is influenced not only by the examined polymorphisms, but also from other point mutations, including hGRαT556I (inactivating mutation of GR, causes Chrousos syndrome), recently reported in a case of adrenal incidentaloma [38]. Strengths of the study are the sample size, which is larger than the sum of all previous published series, and the strict patient categorization.

## Conclusion

In conclusion, the frequency of the different polymorphic GR gene variants does not differ between patients and controls, and this militates against a possible pathogenetic role of the GR SNPs in development of adrenal adenomas. The most interesting result is the association of the N363S SNP with arterial hypertension, which should confer risk susceptibility in patients with non-functioning adrenal adenomas. However, our results demonstrate that the impact of any single SNP on disease expression is limited and their analysis does not provide additional data that may be exploited for patient management.

## References

[1] Szappanos A, Patócs A, Tõke J, Boyle B, Sereg M, Majnik J et al BclI polymorphism of the glucocorticoid receptor gene is associated with decreased bone mineral density in patients with endogenous hypercortisolism. Clin Endocrinol (Oxf). 2009; 71:636–43. 19207316 10.1111/j.1365-2265.2009.03528.x

[2] Manenschijn L, van den Akker EL, Lamberts SW, van Rossum EF. Clinical features associated with glucocorticoid receptor polymorphisms. An overview. Ann N Y Acad Sci. 2009; 1179:179–98. 10.1111/j.1749-6632.2009.05013.x 19906240

[3] van Rossum EF, Lamberts SW. Polymorphisms in the glucocorticoid receptor gene and their associations with metabolic parameters and body composition. Recent Prog Horm Res. 2004; 59:333–57. 14749509 10.1210/rp.59.1.333

[4] Huizenga NA, Koper JW, De Lange P, Pols HA, Stolk RP, Burger H et al A polymorphism in the glucocorticoid receptor gene may be associated with and increased sensitivity to glucocorticoids in vivo. J Clin Endocrinol Metab. 1998; 83:144–51. 9435432 10.1210/jcem.83.1.4490

[5] van Rossum EF, Koper JW, van den Beld AW, Uitterlinden AG, Arp P, Ester W et al Identification of the BclI polymorphism in the glucocorticoid receptor gene: association with sensitivity to glucocorticoids in vivo and body mass index. Clin Endocrinol (Oxf) 2003; 59:585–92. 14616881 10.1046/j.1365-2265.2003.01888.x

[6] van Schoor NM, Dennison E, Lips P, Uitterlinden AG, Cooper C. Serum fasting cortisol in relation to bone, and the role of genetic variations in the glucocorticoid receptor. Clin Endocrinol (Oxf).2007; 67:871–8. 17681029 10.1111/j.1365-2265.2007.02978.x

[7] Di Blasio AM, van Rossum EF, Maestrini S, Berselli ME, Tagliaferri M, Podestà F et al The relation between two polymorphisms in the glucocorticoid receptor gene and body mass index, blood pressure and cholesterol in obese patients. Clin Endocrinol (Oxf).2003; 59:68–74. 12807506 10.1046/j.1365-2265.2003.01798.x

[8] van Rossum EF, Feelders RA, van den Beld AW, Uitterlinden AG, Janssen JA, Ester W et al Association of the ER22/23EK polymorphism in the glucocorticoid receptor gene with survival and C-reactive protein levels in elderly men. Am J Med. 2004; 117:158–62. 15276593 10.1016/j.amjmed.2004.01.027

[9] Trementino L, Appolloni G, Concettoni C, Cardinaletti M, Boscaro M, Arnaldi G. Association of glucocorticoid receptor polymorphism A3669G with decreased risk of developing diabetes in patients with Cushing's syndrome. Eur J Endocrinol.2012; 166:35–42. 10.1530/EJE-11-0722 22048965

[10] Giordano R, Marzotti S, Berardelli R, Karamouzis I, Brozzetti A, D'Angelo V et al BClI polymorphism of the glucocorticoid receptor gene is associated with increased obesity, impaired glucose metabolism and dyslipidaemia in patients with Addison's disease. Clin Endocrinol (Oxf). 2012; 77:863–70. 22587831 10.1111/j.1365-2265.2012.04439.x

[11] Koetz KR, van Rossum EF, Ventz M, Diederich S, Quinkler M. BclI polymorphism of the glucocorticoid receptor gene is associated with increased bone resorption in patients on glucocorticoid replacement therapy. Clin Endocrinol (Oxf).2013; 78:831–7. 23134110 10.1111/cen.12096

[12] Terzolo M, Stigliano A, Chiodini I, Loli P, Furlani L, Arnaldi G et al AME position statement on adrenal incidentaloma. Eur J Endocrinol.2011; 164:851–70. 10.1530/EJE-10-1147 21471169

[13] Mansmann G, Lau J, Balk E, Rothberg M, Miyachi Y, Bornstein SR. The clinically inapparent adrenal mass: update in diagnosis and management. Endocr Rev. 2004; 25:309–40. 15082524 10.1210/er.2002-0031

[14] Morelli V, Donadio F, Eller-Vainicher C, Cirello V, Olgiati L, Savoca C et al Role of glucocorticoid receptor polymorphism in adrenal incidentalomas. Eur J Clin Invest.2010; 40:803–11. 10.1111/j.1365-2362.2010.02330.x 20584071

[15] Zhukouskaya VV, Eller-Vainicher C, Gaudio A, Cairoli E, Ulivieri FM, Palmieri S et al In postmenopausal female subjects with type 2 diabetes mellitus, vertebral fractures are independently associated with cortisol secretion and sensitivity. J Clin Endocrinol Metab. 2015; 100:1417–25. 10.1210/jc.2014-4177 25590217

[16] Young WF Jr. Clinical practice. The incidentally discovered adrenal mass. N Engl J Med. 2007; 356:601–10. 17287480 10.1056/NEJMcp065470

[17] Hamrahian AH, Ioachimescu AG, Remer EM, Motta-Ramirez G, Bogabathina H, Levin HS et al Clinical utility of noncontrast computed tomography attenuation value (hounsfield units) to differentiate adrenal adenomas/hyperplasias from nonadenomas: Cleveland Clinic experience. J Clin Endocrinol Metab.2005; 90: 871–7. 15572420 10.1210/jc.2004-1627

[18] World Health Organization. Preventing and managing the global epidemic of obesity: report of the World Health Organization consultation of obesity Geneva: World Health Organization, 1997. 11234459

[19] Grundy SM, Cleeman JI, Daniels SR, Donato KA, Eckel RH, Franklin BA et al Diagnosis and management of the metabolic syndrome: an American Heart Association/National Heart, Lung, and Blood Institute Scientific Statement. Circulation. 2005; 112: 2735–2752. 16157765 10.1161/CIRCULATIONAHA.105.169404

[20] National Cholesterol Education Program (NCEP) Expert Panel on Detection, Evaluation, and Treatment of High Blood Cholesterol in Adults (Adult Treatment Panel III). Third Report of the National Cholesterol Education Program (NCEP) Expert Panel on Detection, Evaluation, and Treatment of High Blood Cholesterol in Adults (Adult Treatment Panel III) final report. Circulation. 2002; 106: 3143–3421. 12485966

[21] Mancia G, Fagard R, Narkiewicz K, Redón J, Zanchetti A, Böhm M et al 2013 ESH/ESC Guidelines for the management of arterial hypertension: the Task Force for themanagement of arterial hypertension of the European Society of Hypertension (ESH) and of the European Society of Cardiology (ESC). J Hypertens. 2013; 31:1281–357. 10.1097/01.hjh.0000431740.32696.cc 23817082

[22] Expert Committee on the Diagnosis and Classification of Diabetes Mellitus. Report of the expert committee on the diagnosis and classification of diabetes mellitus. Diabetes Care.1997; 20: 1183–1197. 9203460 10.2337/diacare.20.7.1183

[23] American Diabetes Association.Diagnosis and classification of diabetes mellitus. Diabetes Care.2008; 31.

[24] Heinze G. A comparative investigation of methods for logistic regression with separated or nearly separated data. Stat Med. 2006; 25: 4216–4226. 16955543 10.1002/sim.2687

[25] R Core Team (2013). R: A language and environment for statistical computing R Foundation for Statistical Computing, Vienna, Austria URL http://www.R-project.org/.

[26] Koeijvoets KC, van Rossum EF, Dallinga-Thie GM, Steyerberg EW, Defesche JC, Kastelein JJ et al A functional polymorphism in the glucocorticoid receptor gene and its relation to cardiovascular disease risk infamilial hypercholesterolemia. J Clin Endocrinol Metab. 2006; 91:4131–4136. 16849409 10.1210/jc.2006-0578

[27] Damjanovic SS, Antic JA, Ilic BB, Cokic BB, Ivovic M, Ognjanovic SI et al Glucocorticoid receptor and molecular chaperones in the pathogenesis of adrenal incidentalomas: potential role of reduced sensitivity to glucocorticoids. Mol Med. 2013; 18:1456–65. 10.2119/molmed.2012.00261 23196783 PMC3563706

[28] Tzanela M, Mantzou E, Saltiki K, Tampourlou M, Kalogeris N, Hadjidakis D et al Clinical and biochemical impact of BCL1 polymorphic genotype of the glucocorticoid receptor gene in patients with adrenal incidentalomas. J Endocrinol Invest. 2012; 35: 395–400. 10.3275/7840 21738001

[29] Majnik J, Patocs A, Balogh K, Toth M, Gergics P, Szappanos A et al Overrepresentation of the N363S variant of the glucocorticoid receptor gene in patients with bilateral adrenal incidentalomas. J Clin Endocrinol Metab. 2006; 91:2796–2769. 16636127 10.1210/jc.2006-0066

[30] Syed AA, Irving JA, Redfern CP, Hall AG, Unwin NC, White M et al Low prevalence of the N363S polymorphism of the glucocorticoid receptor in South Asians living in the United Kingdom. J Clin Endocrinol Metab. 2004; 89:232–235. 14715855 10.1210/jc.2003-030995

[31] Marti A, Ochoa MC, Sánchez-Villegas A, Martínez JA, Martínez-González MA, Hebebrand J et al Meta-analysis on the effect of the N363Spolymorphism of the glucocorticoid receptor gene (GRL) on human obesity. BMC Med Genet. 2006; 7:50 16725041 10.1186/1471-2350-7-50 PMC1481544

[32] Echwald SM, Sørensen TI, Andersen T, Pedersen O. The Asn363Ser variant of the glucocorticoid receptor gene is not associated with obesity or weight gain in Danish men. Int J Obes Relat Metab Disord. 2001; 25:1563–5. 11673782 10.1038/sj.ijo.0801744

[33] van Rossum EF, Koper JW, Huizenga NA, Uitterlinden AG, Janssen JA, Brinkmann AO et al A polymorphism in the glucocorticoid receptor gene, which decreases sensitivity to glucocorticoids in vivo, is associated with low insulin and cholesterol levels. Diabetes. 2002; 51:3128–34. 12351458 10.2337/diabetes.51.10.3128

[34] Bertalan R, Patócs A, Boyle B, Rigó J, Rácz K. The protective effect of the ER22/23EK polymorphism against an excessive weight gain during pregnancy. Gynecol Endocrinol. 2009; 25:379–82. 10.1080/09513590902730762 19241242

[35] Koper JW, van Rossum EF, van den Akker EL. Glucocorticoid receptor polymorphisms and haplotypes and their expression in health and disease. Steroids. 2014; 92:62–73. 10.1016/j.steroids.2014.07.015 25150015

[36] Ross IL, Dandara C, Swart M, Lacerda M, Schatz D, Blom DJ. 9β Polymorphism of the glucocorticoid receptor gene appears to have limited impact in patients with Addison's disease.PLoS One. 2014; 9:e86350 10.1371/journal.pone.0086350 24466047 PMC3900528

[37] Ross IL, Levitt NS, Van der Merwe L, Schatz DA, Johannsson G, Dandara C et al Investigation of glucocorticoid receptor polymorphisms in relation to metabolic parameters in Addison's disease. Eur J Endocrinol.2013; 168:403–12. 10.1530/EJE-12-0808 23239757

[38] Nicolaides NC, Skyrla E, Vlachakis D, Psarra AM, Moutsatsou P, Sertedaki A et al Functional characterization of the hGRαT556I causing Chrousos syndrome. Eur J Clin Invest. 2016; 46: 42–9 10.1111/eci.12563 26541474

